# Percutaneous fine-needle aspiration for pyogenic liver abscess (3-6 cm): a two-center retrospective study

**DOI:** 10.1186/s12879-020-05239-5

**Published:** 2020-07-16

**Authors:** Shuangjun He, Jie Yu, Hairong Wang, Xuelian Chen, Zhanqiang He, Yi Chen

**Affiliations:** 1 Department of Emergency, South Campus, Shanghai Jiaotong University School of Medicine affiliated Renji Hospital, 2000 Jiangyue Road, Minhang District, Shanghai, 200025 China; 2 Department of Emergency, Shanghai Jiaotong University School of Medicine affiliated Xinhua Hospital, Shanghai, China

**Keywords:** Pyogenic liver abscess, Percutaneous fine-needle aspiration, *Klebsiella pneumoniae*, Ultrasonography, Mortality

## Abstract

**Background:**

The role of ultrasonography-guided percutaneous fine-needle aspiration (PNA) for pyogenic liver abscess (PLA) remains without consensus, especially in abscesses 3 to 6 cm in diameter. The objective of this study was to evaluate the comparative effectiveness and safety of PNA combined with antibiotics.

**Methods:**

This was a retrospective study of patients with PLA that were from 3 to 6 cm in diameter who treated at two medical centers in Shanghai, China, from January 2014 to March 2019. Patients were divided into groups treated by PNA plus antibiotics or antibiotics alone. Patients demographics and clinical data related diagnosis, antibiotic treatment, and patient outcomes were analyzed.

**Results:**

Out of a total of 94 PLA patients, 42 (44.7%) patients received PNA combined with antibiotics, and 52 (55.3%) received antibiotics alone. There were no complications related to PNA. In the PNA group, 13 (31.7%) patients with negative blood culture and 8 (19.5%) patients without blood culture were microbiologically confirmed via aspiration. The time for temperature normalization (*P* < 0.001) and the reduction rate of C-reactive protein within the first week (*P* = 0.031) were significantly lower in the PNA group. In the multivariate analysis, treatment with PNA was more likely to result in clinical improvement of PLA (odds ratio = 0.33, 95% confidence intervals (CI): 0.11–0.96, *P* = 0.043).

**Conclusions:**

PNA combined with antibiotics appears to be a safe, effective, and promising treatment for PLA of 3–6 cm in size. Furthermore, the technique allows for direct microbial sample, which can improve the selection of antibiotics.

## Background

PLA is a potentially life-threatening disease associated with considerable morbidity and mortality. The increasing incidence of PLA reportedly attributable to increases in the frequency of hepatobiliary interventions and the prevalence of hypervirulent *Klebsiella pneumoniae* [1–4]. However, mortality has decreased due to dramatic improvements in diagnosis and treatment methods.

Intravenous broad-spectrum antibiotics are the gold-standard for PLA treatment. However, Webb et al. [5] suggested that antibiotics alone were likely to be insufficient in abscesses > 3 cm. Several studies have shown that a combination of antibiotics and image-guided percutaneous treatment should be considered as first-line treatment for PLA because it achieves an equivalent curative rate with decreased trauma and cost, decreased morbidity, and decreased length of hospital stay [5–14]. However, the standard therapeutic management according to abscess size in PLA patients remains unclear [6, 8, 14–22]. Kulhari and Mandia [19] suggested that percutaneous catheter drainage (PCD) is a better choice as compared to PNA especially in large abscesses that are partially liquefied. They argued that slow improvement in patients treated with PNA is mainly caused by incomplete evacuation of the viscous pus and rapid re-accumulation of pus in the abscess. Some studies advocated drainage in the treatment of liver abscess greater than 3 to 6 cm [6, 8, 20, 23, 24]. PNA is less expensive and less invasive than PCD, and it can be performed in multiple abscess cavities and is free of problems related to catheter care [15, 25, 26]. Zerem and Hadzic [22] endorsed PNA for simple abscesses that were ≤ 5 cm. Singh et al. [27] reported that repetitive PNA and PCD were equally efficient in the management of liver abscesses ≤4.5 cm along the longest diameter.

Few studies have attempted to investigate the role of PNA in PLA of 3 to 6 cm in size. We retrospectively analyzed the patient records of two medical centers to describe the safety and efficacy of PNA for PLA from 3 to 6 cm in size.

## Material and methods

### Patients

The study was approved by the ethics committee of the Shanghai Jiaotong University School of Medicine Affiliated with Renji and Xinhua hospitals. All data were obtained from electronic medical records and reported without personal identifiers.

From January 2014 to March 2019, the medical records of all adult patients who had been discharged with a diagnosis of PLA defined by the ICD-10 diagnosis code K75.0 were reviewed. Data came from two comprehensive tertiary care teaching hospitals in East China (Shanghai Jiaotong University School of Medicine Affiliated with Renji Hospital South Campus and Xinhua Hospital). The diagnosis of PLA was based upon clinical signs and symptoms, medical imaging, and culture and specimen analyses of blood or pus [5].

**Fig. 1:**
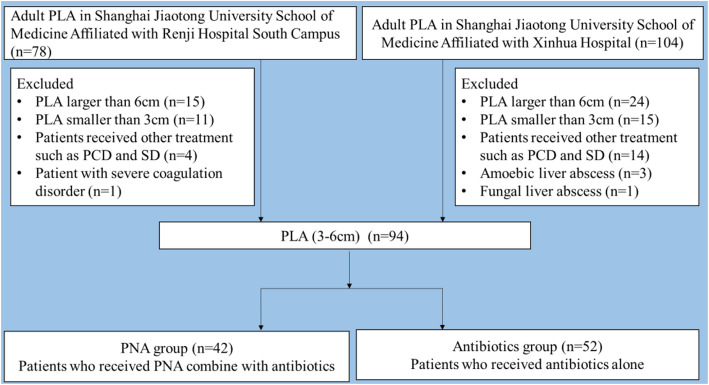
Flow chart of the patients with PLA enrolled in the study during Jan 2014 to Mar 2019

The exclusion criteria in our study were patients with PLA larger than 6 cm or smaller than 3 cm, patients with amoebic liver abscess or fungal liver abscess, patients receiving other treatment, such as PCD or surgical drainage (SD), and patients with severe coagulation disorders. (Fig. 1).

### Data collection and variables definitions

All the clinical data were documented by retrospective chart reviews. Patient variables included demographic characteristics; comorbidities; symptoms at admission and changes during hospitalization; laboratory values and radiological findings.

Hepatobiliary benign disease including fatty liver disease, hepatitis, liver cirrhosis, biliary stone diseases, and biliary tract inflammation. Hepatobiliary malignant disease including hepatocellular carcinoma and cholangiocarcinoma. Abdominal surgery history including cholecystectomy, biliary tract surgery, gastrointestinal surgery, hepatectomy, gynecologic surgery, and pancreatic surgery.

Laboratory values include hematologic, biochemical, and microbiological findings. The radiological values including abscess size, number and location were obtained by the abdominal ultrasonography, computed tomography, or magnetic resonance image.

The Acute Physiology and Chronic Health Evaluation II (APACHE II) and Sequential Organ Failure Assessment (SOFA) score were evaluated retrospectively based on the available data. Data that were not available, such as PaO2, were considered as a zero score.

The complications were defined as sepsis, elevated serum transaminase, elevated troponin, thrombocytopenia, pleural effusion, enteric infection, endophthalmitis and aspiration-related complications (e.g., hemorrhage of any severity), and septicemia. Elevated serum transaminase was defined as beyond the upper limit of normal serum transaminase. Elevated Troponin was defined as defined as beyond the upper limit of normal troponin.

Clinical outcomes included the following: (a) The duration of hospital stay was defined as the number of days of hospital stay; (b) the readmission rate was defined as readmission to hospital with PLA within 60 days; (c) mortality was defined as death during hospitalization; (d) time for temperature normalization (i.e., number of days until the body temperature returns below 37.3 °C since discharge; (e) C-reactive protein (CRP) reduction rate within the first week of hospitalization.

### Antibiotics data

Data on antibiotic use was collected for the duration of intravenous antibiotics, the utilization rate of carbapenems for more than 72 h because of results of microbial sensitivity testing, and the antibiotic use density based on the defined daily dose (DDD) which is determined and updated by the World Health Organization Collaborating Center for Drug Statistics and Methodology [28]. Antibiotic use density was estimated by averaging the antibiotic prescription doses.

### Percutaneous fine-needle aspiration

PNA was performed under real-time ultrasonographic guidance in the ultrasound department using 18G disposable trocar needles of varying lengths (i.e., 10–20 cm). The aspirated pus was drawn into sterile collection vials for aerobic and anaerobic use and then sent for culture and specimen and antibiotic sensitivity testing. We recommend empirical PNA in patients with persistent symptoms, single abscess and mature abscesses, based on assessment by ultrasound specialists.

### Statistical analysis

The continuous variables were presented as means ± standard deviation (SD) or medians (range). Continuous variables were compared using the Student’s t test for normally distributed data or nonparametric tests (e.g., Mann-Whitney U tests) for non-normally distributed data. Categorical variables were reported as numbers (percentage) and were compared using Pearson’s chi-squared test and Fisher’s exact test. Normality was tested using the Shapiro-Wilk test.

The time for temperature normalization was compared between patients receiving PNA and antibiotics-alone. Data were plotted using Kaplan-Meier curves and compared by the log-rank test. For the outcome of normalization of symptoms, a multivariate logistic regression analysis was performed with PNA as a fixed independent variable and statistically adjusted for the presence of carbapenem use, CRP levels on admission, abscess number, and presence of diabetes mellitus. Time to temperature normalization of more than 3 days was considered as the dependent variable in the binary logistic regression to fit the multivariate model. The odds ratios (OR) and 95% confidence intervals (CIs) were estimated in the final model. Differences were considered statistically significant at *P* < 0.05. The statistical analyses were performed using IBM SPSS software version 20.0 (SPSS Inc., Chicago, IL, USA). The graphs were constructed on GraphPad Prism 5.0 (GraphPad Software Inc., La Jolla, CA, USA).

## Results

### Demographic and clinical characteristics

In total, 182 patients with PLA were admitted to the hospitals from January 2014 to March 2019. Of these, 94 patients had a PLA between 3 and 6 cm in size and were enrolled in the study; 53 patients were male, and 41 patients were female. The mean age was 64.2 years (SD 13.3; range 27–89). Patients with and without PNA had similar baseline characteristics (Table 1). The most common comorbidity was diabetes mellitus, which was present in 53 (56.4%) patients. One patient in each group had hepatocellular carcinoma.

**Table 1:** The baseline data, laboratory findings, abscess characteristics, and microbiological characteristics between the groups

	Antibiotics(*n* = 52)	PNA(*n* = 42)	*P* value
**Age, mean ± SD (years)**	65.5 ± 12.2	62.7 ± 14.6	0.314
**Gender, male (%)**	32(61.5%)	21(50.0%)	0.265
**Coexisting diseases, n (%)**			
Diabetes mellitus	26(50.0%)	27(64.3%)	0.165
Hepatobiliary benign disease	15(28.8%)	9(21.4%)	0.412
Hepatobiliary malignant disease	1(1.9%)	1(2.4%)	0.878
Abdominal surgery history	9(17.3%)	11(26.2%)	0.295
**Symptoms at presentation, n (%)**			
Fever	50(96.2%)	38(90.5%)	0.263
Abdominal pain	12(23.1%)	8(19.0%)	0.635
Weakness	16(30.8%)	9(21.4%)	0.308
Nausea/vomiting	17(31.5%)	7(16.7%)	0.076
**Laboratory findings**			
WBC (× 10^9^/L)	9.0 ± 4.3	10.0 ± 3.1	0.183
PLT (×10^9^/L)	155.0(100.8–256.5)	197.5(114.0–303.5)	0.170
CRP (mg/L)	103.0(30.1–160.0)	116.8(75.4–175.8)	0.023
PCT (ng/ml)	2.2(0.3–7.8)	0.8(0.2–14.9)	0.427
GLU (mmol/L)	8.1 ± 2.5	9.5 ± 5.1	0.205
HbA1c (%)	7.6 ± 1.8	7.9 ± 2.5	0.350
**APACHE II score on admission**	4.8 ± 2.0	5.5 ± 3.1	0.161
**SOFA score on admission**	1.1 ± 1.3	1.0 ± 1.3	0.560
**Abscess size(mm)**	40.8 ± 13.2	44.7 ± 9.4	0.112
**Abscess number, n (%)**			0.036
Single abscess	40(76.9%)	39(92.9%)	
Multiple abscess	12(23.1%)	3(7.1%)	
**Abscess location, n (%)**			0.893
Right lobe	43(82.7%)	36(85.7%)	
Left lobe	7(13.5%)	5(11.9%)	
Both lobes	2(3.9%)	1(2.4%)	

Fever (93.6%) was the most common clinical manifestations of PLA in our study, followed by weakness (26.6%), nausea/vomiting (25.5%), and abdominal pain (21.2%). There was no difference in symptoms at presentation, laboratory findings, abscess characteristics, or APACHE II and SOFA scores. However, CRP levels, and abscess numbers were different between the groups.

### Microbiological characteristics

There were 25 (48.1%) patients in the antibiotics alone group and 41 (97.6%) patients in the PNA group who had culture and specimen samples taken. Microorganisms were isolated in the samples of 5 (20.0%) patients in the antibiotics alone group via blood culture, whereas they were isolated in the samples of 32(78.0%) patients in the PNA group via either blood or pus culture (Table 2). More importantly, in the PNA group, there were 13 (31.7%) patients with negative blood culture and 8(19.5%) patients without any blood culture who were confirmed with positive cultures by aspiration (Table 3). The bacteria isolated from the positive cultures were predominantly gram-negative; *Klebsiella pneumoniae* was the most common species in both groups. One patient (3.5%) was polymicrobial among those who had positive cultures from pus samples.

**Table 2:** Microbiological characteristics in the two groups

	Antibiotics (*n* = 52)	PNA(*n* = 42)	
	Blood culture	Blood culture	Pus culture
**Positive**	5(20.0%)	11(34.4%)	29(76.3%)
*Klebsiella pneumoniae*	3	11	24
*Streptococcus spp.*	1	0	1^a^
*Staphylococcus spp.*	1	0	0
*Pseudomonas aeruginosa*	0	0	2
*Escherichia coli*	0	0	2
*Clostridium difficile*	0	0	1^a^
ESBL-Positive	1	3	9
**Negative**	20(80.0%)	21(65.6%)	9(23.7%)
**Unavailable**^b^	27	10	4

^a^There is one patient had polymicrobial infection with *Streptococcus spp.* and *Clostridium difficile in the PNA group*.

^b^Unavailable was defined as blood culture or pus culture was not performed.

**Table 3:** Blood and Pus culture in the PNA group

		Pus Culture			Total
		Positive	Negative	Unavailable	
**Blood Culture**	**Positive**	8(19.5%)	3(7.3%)	0(0.0%)	11(26.8%)
	**Negative**	13(31.7%)^a^	5(12.2%)	3(7.3%)	21(51.2%)
	**Unavailable**	8(19.5%)^b^	1(2.4%)	1	10
**Total**		29(70.7%)	9(21.9%)	4	42

^a^There were 13(31.7%) patients whose blood culture was negative but pus culture was positive.

^b^There were 8(19.5%) patients whose blood culture was unperformed but pus culture was positive

### Antibiotics regimen, complications and outcomes

Details on antibiotics regimens, complications and outcomes associated with the procedure were analyzed (Table 4).

**Table 4:** The antibiotic regimens, complications and outcomes between the groups

	Antibiotics (*n* = 52)	PNA(*n* = 42)	*P* value
**Duration of IV antibiotics (days)**	15.9 ± 8.4	15.1 ± 6.8	0.611
**ESBL positive, n(%)**	1(20.0%)	9 (28.1%)	0.704
**Carbapenems, n (%)**	21(40.4%)	6(14.3%)	0.005
**Antibiotics use density**	211.7 ± 97.5	238.5 ± 90.5	0.174
**Complications, n (%)**			
Sepsis	17(32.7%)	15(35.7%)	0.759
Elevated serum transaminase	5(9.6%)	3(7.1%)	0.669
Elevated Troponin	1(1.9%)	2(4.8%)	0.436
Thrombocytopenia	12(23.1%)	9(21.4%)	0.849
Pleural effusion	0	3(7.1%)	0.050
Enteric infection	0	1(2.4%)	0.263
Endophthalmitis	1(1.9%)	0	0.366
**Aspiration related complications, n (%)**	NS	0	NS
**Outcomes**			
Time for temperature normalization (days)	4.3 ± 2.6	2.7 ± 1.5	< 0.001
Reduction rate of CRP within first week (mg/L·day)	15.4 ± 8.5	21.2 ± 12.0	0.031
Hospital stay (days)	16.1 ± 8.4	15.3 ± 6.8	0.626
Readmission within 60 days, n (%)	3(5.8%)	0	0.114
Mortality, n (%)	1(1.9%)	0	0.263

Carbapenem use was significantly lower in the PNA group than in the antibiotics alone group (14.3% versus 40.4%, *P* = 0.005). However, there was no difference in the duration of intravenous antibiotics or the antibiotic use density.

Orders rates for carbapenems was higher in patients with negative or unavailable culture than in patients with positive cultures. However, the positive rate of extended-spectrum beta-lactamase (ESBL)-producing bacteria was 20.0% (1/5) in the antibiotics alone group and 28.1% (9/32) in the PNA group.

The PNA group was associated with a lower sepsis rate, higher serum transaminase, thrombocytopenia and endophthalmitis rates, and lower readmission and mortality rates than the antibiotics alone group, although the differences were not statistically significant. There were no procedure-related complications, such as hemorrhage of any degree of severity and septicemia in the PNA group in our current study. Only one patient in the antibiotics alone group died of liver failure due to hepatocellular carcinoma.

**Fig. 2:**
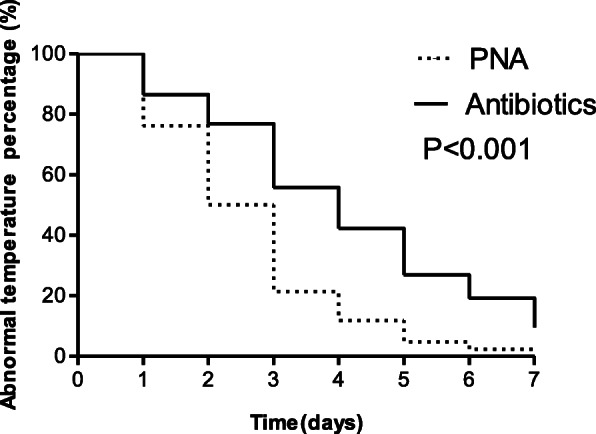
The Kaplan-Meier curves of the time for temperature normalization between groups. Figure shows that the time for temperature normalization was significantly lower in the PNA group than in the antibiotics alone group (*P* < 0.001)

**Fig. 3:**
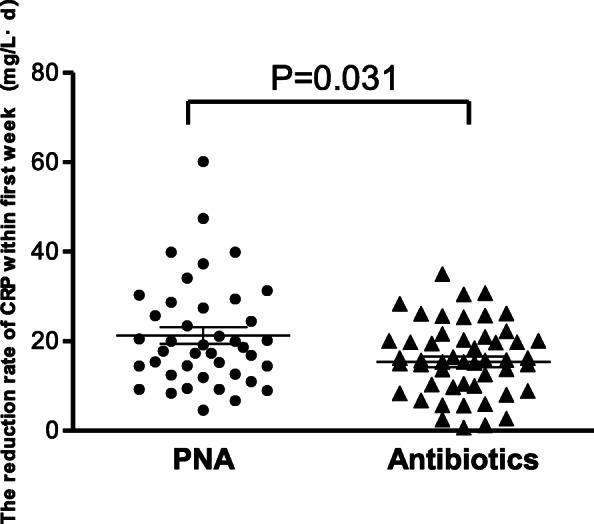
The reduction rate of CRP within the first week between groups. Figure shows that the reduction rate of CRP within the first week was significantly lower in the PNA group than in the antibiotics alone group (*P* = 0.031)

The time for temperature normalization (*P* < 0.001) and the reduction rate of CRP within the first week (*P* = 0.031) were significantly lower in the PNA group than in the antibiotics alone group (Fig. 2 and Fig. 3). However, the duration of hospital stay, readmission rate within 60 days, and the mortality rates were not statistically significant in the two groups. Binary logistic regression analysis suggested that PNA was more likely to be associated with an improvement in clinical benefit (Odds ratio = 0.33, 95% CI: 0.11–0.96, *P* = 0.043) (Table 5).

**Table 5:** Multivariate logistic regression of risk factors for clinical improvement

Variables	OR (95%CI)	*P* value
Percutaneous fine-needle aspiration (yes vs. no)	0.33(0.11–0.96)	0.043
Carbapenems (presence vs. absence)	4.70(1.43–15.37)	0.010
Multiple abscess (presence vs. absence)	0.42 (0.11–1.64)	0.215
CRP on admission (mg/L)	0.99(0.99–1.01)	0.979
Diabetes mellitus (presence vs. absence)	0.85(0.32–2.30)	0.750

## Discussion

This retrospective study compared treatment with PNA combined with antibiotics versus antibiotics-alone for the management of PLA of 3 to 6 cm in size. PNA was more effective and less invasive with fewer complications than antibiotics alone in the treatment of PLA, which was similar to the previous studies [14, 29].

The general baseline characteristics were similar in both groups in our study. However, there were significant differences in the CRP levels, which may indicate that the severity of infection was greater in the PNA group than in the antibiotics alone group. Compared with women, men were more likely to have PLA in our study. In addition, there appeared to be a high prevalence of diabetes mellitus and hepatobiliary disease in our population. Most of the patients in the study had a solitary abscess on the right hepatic lobe, similar to previous reports [30, 31]. We observed that solitary abscesses were more common in the PNA group than in the antibiotics alone group; this difference may be explained by the clinician experience and possibly selection bias. Fever was the most common clinical manifestations of PLA in this study. Since fever is nonspecific, additional imaging examinations were recommended to rule out the possibility of PLA in patients with unexplained fever. This was particularly true in patients with diabetes mellitus and hepatobiliary disease who went to the emergency department. *Klebsiella pneumoniae* was the predominant pathogen of PLA, this result was consistent with other studies [32, 33].

Gao et al. suggested that the normalization of CRP could be used as an independent predictor of the duration of antibiotic treatment after complete percutaneous drainage [34]. Studies have found that withdrawal of the inflammatory stimulus resulted in a marked decrease in the serum CRP concentration similar to first-order elimination kinetics [35, 36]. Notably, the time for temperature normalization and the reduction rate of CRP within the first week were significantly lower in the PNA group than in the antibiotics alone group. Since we do not routinely use antipyretics, so that we could monitor body temperature without missing natural progression of the disease. This difference shows that PNA can effectively reduce the high bacterial load quickly. Although they were nonsignificant, the other clinical outcome measures in this study also favored the PNA group (e.g., duration of hospital stay, readmission rate, and mortality rate). In addition, there were no PNA-related complications observed in our study, which was reassuring. The safety of PNA relies on the skills of an experienced sonographer and strict adherence to the procedure protocol.

The overall mortality rate in our study was lower than in previous studies [37–39]. The low mortality rate may be related to small size of PLA, early antibiotics administration and intervention with ultrasonography-guided percutaneous techniques, the low rate of underlying malignancy. The low APACHE II and SOFA scores may be related to insufficient data and low severity [40].

The early and adequate administration of antibiotics is the cornerstone of PLA treatment. The key to a precise treatment decision is the prompt identification of the cause. We found many patients in the PNA group with either a negative blood culture or without any blood culture, implying that microbial confirmation was from direct sampling of the abscess. PNA appears to be a promising way to collect microbial samples that can guide antibiotic selection. Brunetti et al. found that local cultures of pus are positive more often than blood cultures and that these cultures are essential for an effective treatment plan in immunocompromised patients [41]. Amy et al. reported that obtaining culture pus rather than blood could decrease routine laboratory testing in patients with uncomplicated skin and soft tissue infections [42].

The overuse of antibiotic agents is a public health problem and is associated with increased health care costs and antibiotic resistance [43, 44]. The current study indicates that sample by aspiration may be associated with decreased use of broad-spectrum antibiotics, especially carbapenems. There was no apparent loss of effectiveness from reduced use of broad-spectrum antibiotics even though the duration of intravenous antibiotics and the antibiotic use density did not differ between the groups.

The present study has some important limitations. Relevant missing information that could have influenced the outcome may have been excluded from the analysis and it was not feasible to obtain all the data because this was a retrospective study. Most patients were treated empirically with antibiotics and did not receive precise treatment for their particular organism. A prospective study that addresses limitations and that avoids selection bias is underway at our institution. If those results align with these current findings, they are likely to have an economic impact as well as an improvement in the quality of life of PLA patients.

## Conclusion

PNA combined with antibiotics is a treatment that has potential to be a first-line approach in the treatment of PLA of 3 to 6 cm in size. Our study highlights PNA as a method that improves the rate of positive cultures to guide the selection antibiotics.

## Data Availability

The datasets used and/or analyzed during the current study are available from the corresponding author on reasonable request.

## Abbreviations

PLA: Pyogenic liver abscess
PNA: Percutaneous fine-needle aspiration
PCD: Percutaneous catheter drainage
OR: Odds ratio
95% CI: 95% confidence interval
US: Ultrasonography
CT: Computed tomography
MRI: Magnetic resonance image
SD: Surgical drainage
APACHE II: Acute Physiology and Chronic Health Evaluation II
SOFA: Sequential Organ Failure Assessment
DDD: Defined daily dose
CRP: C-Reactive protein
WBC: White blood cell
PLT: Platelet
PCT: Procalcitonin
GLU: Glucose
HbA1c: Glycosylated hemoglobin
ESBL: Extended-spectrum beta-lactamase
